# Analysis of business models for delivering energy efficiency through smart energy services to the European commercial rented sector

**DOI:** 10.12688/openreseurope.15240.1

**Published:** 2022-11-28

**Authors:** Luciano De Tommasi, Sotiris Papadelis, Ruchi Agrawal, Padraig Lyons

**Affiliations:** 1 International Energy Research Centre, Tyndall National Institute, University College Cork, Lee Maltings, Dyke Parade, Cork, T12 R5CP, Ireland; 2 Hebes Intelligence, Filippoupoleos 36, Athens, 10443, Greece

**Keywords:** Energy efficiency, smart energy services, split-incentive issue, commercial rented sector, Energy Service Companies

## Abstract

In this paper, we perform a comparative analysis of business models used by Energy Service Companies (ESCOs), suitable for the deployment of energy efficiency measures in the commercial rented sector across Europe. These models can effectively contribute to solving the split-incentive issue that arises in the rented building scenario. Some of them are obtained from their “traditional” counterparts, which do not consider the rented scenario, but just a bipartite agreement between an Energy Service Company and its client. The EU Horizon 2020 project SmartSPIN (Smart energy services to solve the SPlit INcentive problem in the commercial rented sector) targets delivery of enhanced energy services for commercial rented sector. These enhanced energy services (a) combine demand management services and energy efficiency interventions, (b) facilitate the adoption of renewables, (c) optimize the balance between demand and supply, (d) alleviate the split incentive issue. The pilot implementation of project will be performed in a business park in Greece, in an office building in Ireland and in two shopping centers in Spain. Key recommendations toward the implementation of such a smart energy service are provided in this paper. They have been obtained from a detailed analysis of ten interviews of key stakeholders of the energy efficiency section and of the commercial rented sector, along with an analysis of a selection of the most relevant technical literature.

## 1. Introduction

The split incentive issue is a key challenge to the deployment of energy efficiency measures in commercial rented buildings across Europe ( Castellazzi *et al*., 2017). This situation arises because those parties who pay the energy bills (and that enjoy the energy savings) are different from those who make investment decisions. In ( Nie *et al*., 2020) two types of split incentive are considered: investment split incentives and behaviour split incentives. The former may hinder investment decisions in energy efficiency measures, whereas the latter may hinder behaviours which determine a lower energy consumption. The split incentives in the U.S. commercial rented market were analysed in ( Jessoe *et al*., 2020). It was found that 20% of tenants rent space in commercial buildings with electricity included into their monthly rent. Such a type of contract structure determines an incentive to over-consume energy (behavioural split-incentives) because electricity’s marginal price is zero for tenants. The remaining 80% of tenants pay their own monthly utility bills, which will reduce the incentive for building owners to invest in energy efficiency measures, if they cannot increase the rent premium to fund the interventions to improve energy efficiency (investment split-incentives). The four possible cases with respect to split-incentives in the commercial rented sector are summarized in Table 1. Furthermore, the fact that tenants do not know for how long they will rent the current commercial units is creating an uncertainty regarding the rent income for the landlords, which may affect the decisions of investing in energy efficiency measures. The issue is sometimes referred to as temporal split incentive ( Bird & Hernández, 2012).

**Table 1.** Types of split incentives for the commercial sector.

	Occupants can invest in energy efficiency technology (owner)	Occupants can’t invest in energy efficiency technology (renter)
Occupants pay the energy bill	No split incentives	Investment split incentives
Occupants do not pay the energy bill	Both investment split incentives and behaviour split incentives	Behaviour split incentives

It is worth noting that works in a rented space require the landlord's consent, and a tenant can be reluctant to have any dealings with the landlord than strictly necessary, therefore in most cases a tenant will not ask the landlord energy efficiency improvements ( Barton, 2014). Green leasing is the main instrument for both landlords and tenants to collaborate for achieving the common objective of improving energy efficiency and reducing CO2 emissions, and it can be considered as the first step toward overcoming the split-incentive issue ( SmartSPIN D3.3, 2022). In a green lease, either the landlord or the tenant might assume a paternalistic role, wishing to force at some extent the other party to collaborate for achieving certain "green" goals, whereas a better-balanced relationship would enable to set out mutual objectives in the lease in a collaborative manner ( Brooks *et al*., 2008).

A new business model is required to overcome the split incentive issue in the commercial sector in Europe and to unlock the opportunities for increasing energy efficiency and flexibility of many commercial buildings. A well-designed business model might trigger a much greater uptake of smart energy services deployed *via* performance-based contracting throughout the commercial sector. The approach followed in this paper toward establishing an ESCO business model addressing the split incentive issue in the commercial sector, is the analysis of the traditional ESCO business models, which do not consider the rented situation and their extension to the rented scenario. These models are all based on the concept of Energy Performance Contracting (EPC; European Commission, 2022), in which the ESCO guarantees that the package of energy efficiency measures delivered to the client will generate sufficient reductions in the energy bill of the client to repay the initial capital investment within the duration of the EPC. The EPC requires a robust measurement and verification (M&V) methodology to verify that the predicted savings are obtained and includes some penalty clauses to compensate the client when the agreed savings are not obtained. The M&V process must be precise and unbiased to create the trust required to sign an EPC contract and to avoid contractual disputes ( Agenis-Nevers *et al*., 2021). With the performance guarantee model, the ESCO does not receive payment unless they deliver the agreed energy or cost savings. Different types of EPC exist, and they are linked with the underlying business models. Most common are Shared-savings, Guaranteed savings, Energy cost-trust, and Finance lease, which determine the division of the savings between the ESCO, and its client and the service delivered ( Qin *et al.*, 2017; Shang *et al.*, 2017). They are summarized in Table 2.

**Table 2.** Business models based on the Energy Performance Contracting concept.

N.	ESCO traditional model	Description
**1**	Shared Savings Model	The ESCO assumes both the performance and the credit risk throughout the EPC project. The ESCO and its client share the energy savings resulting from the project according to a previously agreed proportion. The ownership of the energy-efficient equipment is transferred to the ESCO’s client at the end of the project; thereafter, the obtained energy savings entirely accrue to the ESCO’s client.
**2**	Guaranteed Savings Model	The ESCO assumes the performance risk but not the credit risk. The ESCO’s client takes the responsibility to finance the EPC project through banks, other investors or using own resources. The ESCO guarantees a certain level of energy savings. If the actual savings are lower than the guaranteed threshold, the ESCO will pay the difference to its client. On the other hand, if the actual savings are higher than the agreed threshold of guaranteed savings, the excess of energy savings will be shared between the ESCO and its client according to a previously agreed proportion.
**3**	Energy Cost Trust Model or Chaffee Model	This model is a type of energy supply contracting where the ESCO takes over the energy supply of its client. The client pays a fee to the ESCO which may be discounted from the energy bill. If the bill exceeds a certain threshold the ESCO will compensate its client for the excess part of the payment. At the end of the EPC contract period, the ESCO’s client earns all the energy savings.
**4**	Finance Lease Model	The ESCO requests financing from a finance lease company offering the future energy savings of the EPC project as a guarantee. The finance lease company provides the energy efficiency equipment to the ESCO for the EPC project. The financier company owns the equipment throughout the EPC duration. The ESCO’s client pays for the equipment using energy cost savings following a previously agreed timetable. At the end of the EPC project, the client will own the equipment.

The H2020 SmartSPIN project aims to remove the barrier of the split incentive through an innovative business model that couples the contractual agreements between tenants, building owners and energy efficiency providers with technologies for energy monitoring, management and measurement and verification (M&V). To increase transparency, credibility and persistence of savings, a new set of tools should be developed with the contribution of the whole value chain, including stakeholders of both the supply side (ESCOs, M&V specialists, etc.) and the demand side (tenants, building owners *etc*.).

In June 2020, BASE, AGORIA, ANESE and Innoenergy in Belgium, the Netherlands and Spain using Horizon 2020 funding launched an Efficiency as a Service (EaaS) initiative. The project proposed a pay-per-use model allowing the end-customers to pay only for the service received, rather than the physical infrastructure or product required for its delivery. The costs of the installation and maintenance of the equipment delivering energy efficiency is recovered through periodic customer payments. The customer pays fixed-cost-per-unit of the energy efficient service used, such as euros per hours of lighting, cubic meter of compressed air, per ton of refrigeration ( Efficiency as a Service Initiative, 2020). SmartSPIN will extend such approach to the commercial rented sector, overcoming the split-incentive issue and delivering EaaS to the rented properties.

The development of an enhanced EaaS business model requires the analysis of the potential opportunities for energy efficiency, flexibility, and renewable energy deployment in different commercial building types ( *e.g*., office, shopping center, sports facility *etc*.) as well as of future market developments such as dynamic tariff structures and peer-to-peer trading ( Kessels *et al*., 2016; Tushar *et al*., 2020). Moreover, it requires the analysis of energy and non-energy benefits such as: (a) energy related payments such as tax incentives, feed-in-tariffs, reduced energy bills, demand response revenues ( Eid *et al*., 2016) and (b) non energy benefits such as increased building value, increased rental value, increased occupant comfort, greater productivity, improved brand image ( Edwards, 2006; Myers *et al*., 2007).

An initial service model was investigated, involving both landlords and tenants to deliver different packages of energy efficiency measures which can generate energy savings ( SmartSPIN D2.4, 2022). The installation of no-cost and low-cost measures (that does not require permission from the landlord to perform substantial upgrade works) enables to accumulate energy savings which can be used by an ESCO to finance (partially or totally) more advanced energy conservation measures ( Agrawal *et al*., 2022). The payment of a monthly, quarterly or annual fee by the tenant (that is linked, directly or indirectly, to the energy savings achieved on their utility bills) represents the revenue stream for the ESCO. This revenue stream can be used to reward the landlord according to the specific business model adopted, such as shared savings, guaranteed savings, combination of shared/guaranteed savings, Chaffee model, *etc*. ( SmartSPIN D2.1, 2022; SmartSPIN D2.2, 2022).

## 2. Methods

Traditional EPC business models already available in the published literature where an ESCO enters into an agreement with a client to implement energy efficiency measures were thoroughly analyzed. These models were adapted to the rented case to figure out how three subjects (landlord, tenant, ESCO) might enter into an agreement for improving energy efficiency, successfully overcoming the barrier of the split-incentive ( SmartSPIN D2.2, 2022). Furthermore, two more recent business models applicable to the rented sector were considered: the Managed Energy Service Agreement and the Metered Energy Efficiency Transaction Structure ( SmartSPIN D2.1, 2022). Ten interviews with relevant stakeholders were carried out by the SmartSPIN project ^1^ ( SmartSPIN D2.1, 2022; SmartSPIN D2.4, 2022). The goal of these interviews was to determine the challenges and drivers for the promotion of energy efficiency and smart energy services, and to draw a list of recommendations for an innovative energy service for the commercial rented sector. The profiles of the stakeholders selected for an interview are:

1.   A well-established and known energy agency. It aims at accelerating the low-carbon transition of a European capital to mitigate the effects of climate change and improve the lives of citizens.

2.   An ESCO and EPC facilitator specialized in realizing comfort, energy efficiency and renewable energy in existing non-residential buildings, industrial plants and apartment buildings.

3.   An energy and carbon consultancy working with both public and private sector organizations to deliver sustainable solutions.

4.   A national association of ESCOs, which counts more than 120 members, specialized in energy services, technologies, and investments.

5.   A real estate investment trust focusing on shopping centers’ assets, which are owned and managed by the company. The company’s activity is well established in 16 European countries (Belgium, France, Scandinavian countries, Germany, Netherlands, Poland, Greece, Portugal, and Spain).

6.   An organization that aims at promoting the metered energy efficiency transaction structure (MEETS) model.

7.   A Spanish energy service company established in 2007 that offers services for engineering and energy assistance, Industrial work and installations, maintenance, energy services, and R&D.

8.   A company providing essential service to the public sectors like defense, transport, justice, immigration, healthcare and other citizen services across their four operating regions UK and Europe, North America, Asia Pacific and Middle East.

9.   A very specialized ESCO based in Ireland. It specializes in managing and intelligently automate power-matching transactions on a local level.

10.   An expert from the European Commission

The validation of final SmartSPIN business model will be performed at the pre-selected demonstration sites in Spain, Greece and Ireland during the project.

Stakeholders provided written informed consent and ethical approval was granted by Social Research and Ethics Committee of University College Cork (approval number: 2021–135) prior to interviews taking place.

## 3. Business models for the commercial rented sector addressing the split-incentive issue

### 3.1 Equipment lease

**Figure 1.**
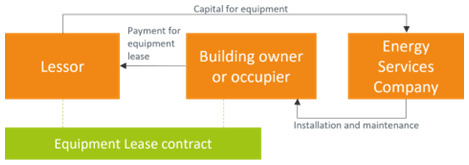
Equipment lease business model.

An equipment lease is a contract signed between two parties, the owner of the asset and the user of the asset, which gives the right to the user to use the asset for a specific period, against a fixed amount paid to the owner of the asset ( SmartSPIN D2.1, 2022). The equipment lease business model is represented in Figure 1. Relevant examples are the solar-as-a-service (SaaS) model and the heat-as-a-service (HaaS) model. With the SaaS, the ESCO leases PV panels and is responsible for financing, installation and maintenance, offering a solar energy tariff and dealing with energy export agreements. The HaaS delivers heat with a certain level of comfort agreement and can work with district heat network or with heat pumps, with ESCOs leasing the infrastructure ( Brown *et al*., 2022). In most cases, the service provider assumes the financial and the technical risk, which incentivizes routine maintenance of the equipment. In some variations of the model, the service provider couples the offering with performance guarantees. It is possible that the building owner finances the equipment installations; either the building owner acts as a lessor or the building owner is the one to get into a contact with a lessor. In this case, a pass-through clause is included into the landlord-tenant lease to allow the costs of the efficiency measures to be passed through to the tenant as incremental upcharge. It is worth noting that a power purchase agreement (PPA) may be a better alternative to an equipment lease if the building owner does not want to be responsible for ongoing maintenance costs ( Alternative Energy Ireland, 2022). Leasing may increase corporate performance measured in terms of market value because investors generally consider it as a business model that can contribute to increase the company's value ( Ionaşcu & Ionaşcu, 2018).

### 3.2 Shared savings business model

**Figure 2.**
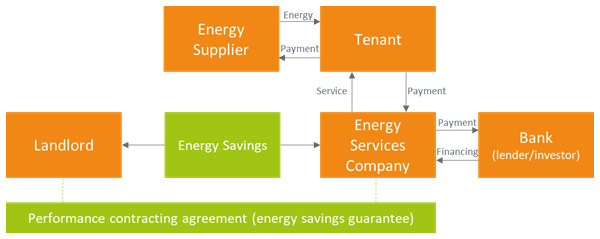
Shared energy savings business model in the rented case.

With the shared savings business model, the ESCO shares the savings with the landlord ( Figure 2). Both ESCO and landlord obtain a revenue stream from energy efficiency service delivered by the ESCO. The ESCO is responsible for designing, financing, and implementing the energy efficiency project, usually obtaining a fixed portion of the savings over a fixed period. The ESCO is also responsible for the verification of the savings during the contractual period. The tenant pays the fees for the energy efficiency service to the ESCO and enjoys non-energy benefits such as a renewed premise and lower carbon-dioxide emissions. The risks for the landlord related to the implementation of the energy efficiency projects are limited. The tenant also pays their own utility bills to the energy supplier. This is the most energy efficient solution, since it was found that the firms that pay their own utility bills consume about 3 percent less electricity annually than those ones whose utility bills are included into rents ( Jessoe *et al*., 2019). Furthermore, energy savings may or may not be shared with the tenant, depending on the amount of reduction of the monthly energy bill with respect to the payment for the ESCO service (sharing of savings with the tenant are not shown in the Figure).

### 3.3 Guaranteed savings business model

**Figure 3.**
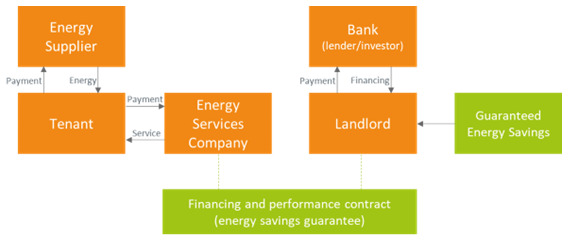
Guaranteed energy savings business model in the rented case.

The ESCOs may also be offering an energy efficiency service guaranteeing a stable amount of energy savings (which in turn would determine a stable revenue stream if energy prices were constant) to the landlord (guaranteed energy savings, Figure 3). In such a case the ESCO is still responsible for designing and implementing the energy efficiency project but would leave the responsibility for financing it to the landlord. Also in this case, the tenant pays the fees for the energy efficiency service to the ESCO and a reduced bill to the energy supplier, thanks to the installation of energy efficiency measures. The landlord may secure a stable revenue stream at the price of having to directly liaise with a bank or another investor for the financing of the energy efficiency measures.

**Figure 4.**
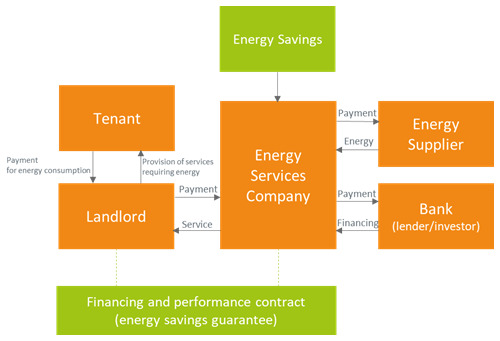
Chaffee business model.

**Figure 5.**
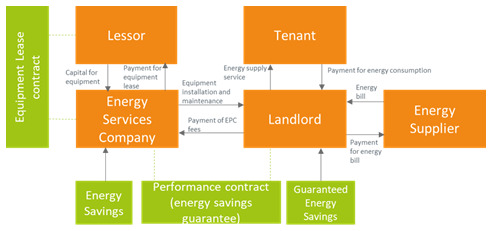
Finance lease business model.

### 3.4 Chaffee business model

An ESCO may offer a complete energy service including both energy supply and energy efficiency to a commercial rented facility. This business model is sometimes referred in the literature as Chaffee model ( Figure 4). In such a case, the ESCO is responsible for the operation and maintenance of the entire energy system of its customer. The ESCO has the opportunity of securing a substantial revenue stream getting all the savings if contractual targets are met. To achieve such goals (which are specified in the contract), the ESCO needs to manage and transform the customer’s energy system and will self-finance the related upgrade projects. If the targets are not met, the ESCO will pay a compensation, which depends on the energy savings shortage. The landlord has a contract with the ESCO and pays for both energy provision and the energy efficiency. Rather than a revenue stream from the energy efficiency, the landlord enjoys a thorough energy service from the ESCO with favorable contractual conditions. Moreover, the landlord receives a payment from the tenant(s) for the energy expenses due to the energy consumption of the tenant.

### 3.5 Finance lease business model

With the finance-lease business model ( Figure 5), the energy savings of the EPC project taken by the ESCO as a guarantee allow ESCO to request financing from a lease company (lessor). The lessor will invest in the EPC project providing the necessary equipment to the ESCO, while retaining ownership of the equipment throughout the contract duration. The payments for the equipment are performed using the energy user's cost savings according to an agreed timetable. The ownership of the equipment is transferred to the energy user at the end of the contract. The main advantage for the ESCO is that lease payments are usually lower than loan payments, because depreciation and interest expenses associated with the purchase of the equipment should not apply ( Weiss, 2003). Moreover, through the finance lease contract, the ESCO transfers the credit risk to the finance lease company ( Qin *et al*., 2017).

### 3.6 Energy efficiency as a service business model

**Figure 6.**
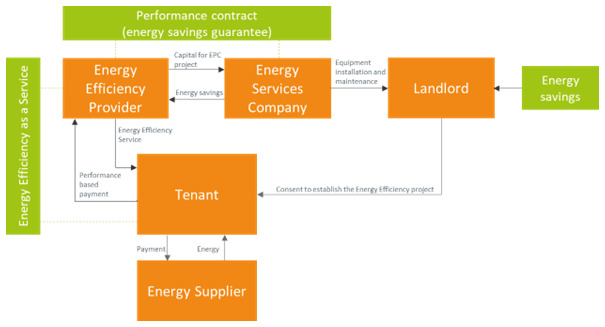
Energy Efficiency as a Service business model.

The energy efficiency as a service (EEaaS) business model is based on the idea that the promotion and up-scaling of energy efficiency requires treating all relevant costs as operational costs. The rationale is that buildings are assets and most building owners have already borrowed against them. As a result, the balance sheets of the building owners are already too crowded to add new liabilities for energy retrofit capital. In its most basic form, the EEaaS model has the structure of Figure 6. The EEaaS provider has an EPC with performance guarantees agreement with an ESCO/contractor, while receiving payments from the building user according to the achieved energy savings. Pay-for-performance rules and transactions govern the relationships of all the involved parties in the model. Since energy efficiency is determined by the characteristics of the equipment as much as by the way it is operated, it makes sense to link the consumers’ payments to the overall performance of the service, quantified as the difference between the actual energy consumption and the energy consumption had the relevant intervention and optimizations not taken place. This is particularly relevant when the energy retrofit includes upgrades for improved monitoring and control of the systems’ operation. EEaaS models incentivize active management and optimization: the greater the performance of the service, the higher the added value for the consumer and the payments to the service provider. With EEaaS, the energy efficiency provider owns the assets delivering energy efficiency; therefore, there is no assets or liability added to the building owner’s balance sheet ( U.S. Department of Energy, 2022b).

### 3.7 The managed energy services agreement model

**Figure 7.**
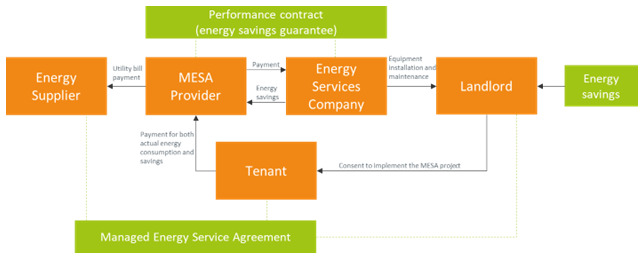
The Managed Energy Service Agreement business model.

An energy service agreement (ESA) is a variant of EPC that provides integrated financing of energy saving measures along with a long-term performance guarantee. A Managed Energy Services Agreement (MESA) model integrates the ESA model providing the energy saving improvements with the EaaS model providing the final energy service ( Brown *et al*., 2022). Under a MESA agreement, the MESA provider acts as an intermediary between the consumer and the utility by assuming the responsibility for the utility bills and charging the customer for both the actual energy consumed and the estimated energy savings due to the energy efficiency measures. In rented buildings the service provider directly passes the charges through to tenants ( Figure 7). Since the EEaaS providers pay for and own the equipment, they face the risk of the building being left without tenancy. One way to mitigate this risk is by treating an energy efficiency upgrade as an option that has both a cost to acquire (the cost of the upgrade) and the capability to produce value when utilized by the tenant. When the model is operational, the added value that is generated for the tenant should cover the cost to acquire. In the case of tenancy interruption, the building owner must have agreed to pay to the provider a minimum fee for making the efficiency upgrade available in the first place.

### 3.8 The metered energy efficiency transaction structure

A metered energy efficiency transaction structure (MEETS) model consists of the following transactions ( Egnor *et al*., 2016):

•  The building owner offers the building spaces and functions for the installation of the energy efficiency measures.

•  The MEETS service provider pays for and maintains the measures in the building, and in return, has a long-term agreement to exploit the value of the energy savings. MEETS uses the term *energy tenant* to highlight the acquired right to harvest the added value of the energy efficiency upgrades. For this right, the service provider pays the building owner rent for using the site. These payments are an additional rental income for the building owner.

•  The energy tenant delivers to the utility the yield from the metered energy efficiency (energy savings due to the upgrades).

•  The utility bills the building, at retail, for both actual consumption and metered efficiency. As in the on-bill-repayment case, the utility is actively involved by offering its billing system for the charging the tenants and redistributing the value to the service provider.

The main reason for utilities to participate in a MEETS scheme is the opportunity to buy energy savings (like a PPA for energy efficiency) and comply with energy efficiency obligations that are imposed on them in the framework of Article 7 of the Energy Efficiency Directive.

**Figure 8.**
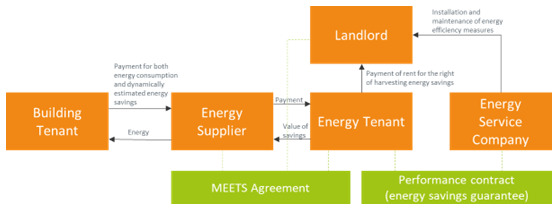
The Metered Energy Efficiency Transaction Structure.

•  Part of the financial benefits can be used for incentivizing the tenants to avoid behaviours that lead to the deterioration of the energy efficiency measures.

•  The building owner treats the energy efficiency improvements the same way other conventional tenant improvements are treated. At the conclusion of the agreement with the service provider, the improvements become property of the building owner, free of debt or other financial liability. The MEETS model is summarized in Figure 8.

An important observation from MEETS initiatives in the USA is that third-party lenders are willing to engage only if the utility is a proper counterparty, instead of it just passing through the collected payments for metered efficiency. In other words, the utility should treat consumption and metered efficiency in the same way: if any of the respective parts of the bill are not paid, the utility has the right to stop the energy supply to the building.

## 4. Recommendations for an enhanced energy service in the commercial sector

The recommendations drawn from the ten interviews with key stakeholders (introduced in section 2) and literature review are summarized in Table 3. As a next step, they will be used as input for the SmartSPIN service definition and tailored to the three demonstration sites of the project in Spain, Greece, and Ireland. This process will also enable to elaborate on their practical applicability and adaptation to different types of rented commercial buildings.

**Table 3.** Recommendations for SmartSPIN Service.

N.	Recommendation	Why	Barriers removed	Supporting evidence
1	Determine the key features of the SmartSPIN service and how these are deployed including whether an EPC or another contractual agreement (this depends on the specific feature and on the country). A package of energy efficiency measures can be installed without complicating the building management practice or add risk to schedule and budget.	Forthcoming SmartSPIN service definition (D3.4) will list the key features of the service definition and state the conditions under which each feature should be deployed	Ambiguity related to deployment of SmartSPIN features in different countries	Interview 1. SmartSPIN D2.2 and D2.3. Literature ( Mathew *et al*., 2020).
2	If a smart energy service is delivered by means of an EPC, consider the involvement of an EPC facilitator when engaging with landlord and tenants. A Facilitator can facilitate the communication between all stakeholders involved in the project and secure interdisciplinary project management capabilities.	Facilitators can help to sell more SmartSPIN services and deliver all the features in the various countries	High complexity of EPC implementation	Interview 1. Literature ( Bleyl *et al*., 2013). Activities and training for EPC facilitators in Ireland (SEAI)
3	The Energy as a Service (EaaS) model should be used to deploy the SmartSPIN service if EaaS applies to the features delivered to a specific client and it is the most cost-effective solution.	The EaaS model is a comprehensive Energy Contract including 5 benefits: 1. Energy management 2. Maintenance 3. Total guarantee of the equipment 4. Improvement works 5. Improvement of energy efficiency EaaS might be the best solution for rented buildings.	Reduced cost effectiveness of uncoordinated contractual solutions for energy management, maintenance, equipment guarantees, improvement works, Improvement of energy efficiency. Market and behavioural barriers that prevent consumers to upgrade to more energy efficient technologies and act in their own self-interest.	Interview 4. Technical literature ( Cleary & Palme, 2019).
4	Standardise the M&V process applicable to the features of the SmartSPIN service. Ensure that measurement uncertainty is quantified and that the achieved accuracy allows identifying significant changes in energy consumption. Invest no more than 10% of the annual project monetary savings in M&V but make sure that M&V costs are not decreased up to the point where the data lose all their value. In addition, the M&V task should be carried out by a third party (different from the ESCOs implementing the measures) so that no conflict of interests is present.	Clients subscribing the SmartSPIN service must be well informed about the M&V process and fully trust it in order to avoid disputes on the actual energy savings achieved. Fear of measurement errors were reported in one of the interviews conducted in WP2 (interview 5). Debates regarding achieved energy savings may lead to court cases and failure of projects.	Lack of information about M&V and lack of trust in ESCOs	Interview 5. Literature ( Boza-Kiss *et al*., 2015; Clean Energy Ministerial, 2014).
5	Develop a service that requires the engagement of both landlord and tenants. Determine which features of the SmartSPIN service involve only landlord and which ones only tenants and which ones both.	SmartSPIN aims to exploit all the opportunities to improve energy efficiency in the commercial rented sector such that the energy and non-energy benefits are maximised for all the parties involved. Building’s occupants can control up to 80% of energy use in a commercial building.	Simplification of the rented scenario that may lead to incomplete exploitation of the potential for energy efficiency improvements in a commercial building.	Interview 4. Study of U.S. Department of Energy, 2022a.
6	Determine which features of the SmartSPIN service are best delivered using an EPC and which ones are best delivered using PPAs, equipment leasing, EaaS. Building owner should understand differences between contracts before determining which the best fit is.	EPC may be more complex and difficult for users to understand contract clauses. Singing an EPC requires a lot of time, approximately 9 moths, because of the different actors involved and their different expertise.	Difficulties in getting an EPC agreed by the potential clients of the commercial sector.	Interview 4. Study of U.S. Department of Energy, 2022b.
7	Consider as features of the SmartSPIN service: energy management, changing the electricity supplier, equipment installation/RES (solar PV installations) or replacement and O&M services. Energy efficient refurbishment uses a package of measures that deliver a net saving over 15 years.	A combination of several features in one SmartSPIN service will likely enable to maximise the benefits for landlords, tenants and ESCOs.	Potential lack of coordination between different energy efficiency interventions	Interview 4. Literature ( IPF Report, 2017).
8	The SmartSPIN service should improve tenants’ efficiency (not only the energy efficiency of the complete commercial building). Tenants can be engaged with appropriate strategies increasing general energy awareness, and incentivizing reduction in energy consumption.	Tenants that are informed about buildings’ energy efficiency and energy rating are willing to pay a higher rental rate for improved energy efficiency. If tenants pay less energy fees, they might be able to pay an even higher rental rate to the building owner.	Split-incentive between landlord and tenants	Interview 5. Best practices ( ENERGY STAR Commercial Buildings Program, 2022a). ( Melvin, 2018) on “asymmetric information causes market distortion”.
9	The use of submetering in commercial buildings is recommended for fair billing. Issue energy consumption invoices to the tenants based on the readings of the meters.	Meters are often not used in all cases and the payments of the tenants are proportional to the sqm.	Inaccurate billing of energy consumption of tenants.	Interview 5. The study ( Bennet, 2017).
10	Determine a tariff for the electricity, gas consumption and water used by the tenants, which is independent of the season.	Landlord to tenant electricity supply is profitable for landlords (using PVs or CHPs), while at the same reducing tenants’ electricity bills. Billing of tenants’ consumptions must be fair and reflect their own utilization of electricity, natural gas and water. Differences in the energy prices during the different seasons of the year are difficult to explain to the tenants.	Unclear/unfair tariff applied to tenants.	Interview 5. Article ( BMWK, 2022). The paper ( Baum *et al*., 2018).
11	The SmartSPIN service and its deployment must be tailored to the specific relationship between landlord and tenant distinguishing the cases where 1. The landlord owns everything and 2. The landlord just owns the space. Tenants and landlords must work together toward the goal of energy efficiency.	If the landlord is in control of everything, the ESCO should better engage with landlord. If tenants are allowed to install their own equipment the ESCO will engage with them.	Uncertainty regarding how an ESCO should engage with landlord/tenant	Interviews 3 and 5. Article ( ENERGYSTAR, 2022b).
12	Determine the revenue stream model that best suits the SmartSPIN service among those ones shown in D2.2, considering the specificities of the country where the service is going to be deployed.	Revenue streams must satisfy the expectation of the parties for the actual deployment of the service.	Uncertainty regarding the revenue streams associated with the SmartSPIN service.	SmartSPIN D2.2. Scientific literature on business models for ESCO services ( Qin *et al.*, 2017; Shang *et al.*, 2017).
13	Consider feed-in tariff and Dynamic tariff structures in service definition if relevant with the country where the SmartSPIN service is going to be deployed in future.	Feed-in tariff and dynamic tariff will strengthen the business case for the SmartSPIN service.	Assumptions of no feed- in tariff and standard flat rate for electricity or day/ night rate	Interview 3. SmartSPIN D2.2 and D2.3. Technical literature, e.g. ( Boait, 2009).
14	The SmartSPIN service must provide a full package of measures including energy management activities, installing the equipment and sensors, monitoring energy, giving advice on the energy consumption, monitoring the project’s implementation and performance.	The SmartSPIN service is expected to advance the state-of-the-art of the ESCO services in order to be more attractive for the commercial rented sector.	Lack of information about ESCO services and lack of trust in ESCOs.	Interview 3. D2.2 and D2.3. Technical report ( Suruhanjaya Tenaga, 2016). Paper ( Moreno *et al.*., 2014).
15	Consider the contractual arrangements allowing delivering high-energy efficiency for a wide range of project sizes.	Increase the opportunities to deliver high- energy savings for all the clients aiming at achieving that regardless of their size.	Only clients of certain sizes may obtain rewards from energy efficiency interventions.	SmartSPIN is not tied to a specific type of EPC but will develop its own contractual template to implement the SmartSPIN service.
16	Develop a service that can delivered to Energy Communities (ECs) including commercial rented units and that may allow energy communities to operate as aggregators of flexible energy resources. Consider that ECs might purchase energy collectively. Consider that collective investment and supply from renewable energy is a main goal for EC in Greece, whereas collective self-consumption is a main goal in Greece and Spain.	The number of energy communities is increasing in several EU countries (Ireland is one of them) and the split-incentive issue has not been addressed within energy communities yet.	Lack of knowledge about business models that can solve the split incentive issue in energy communities	Interviews 7 and 9. The report ( EC Bridge, 2021)
17	An Energy Services Agreement may cover one or more services such as Energy analysis and audits; Project identification and appraisal; Project design and implementation; Energy management; Property/ facility management; Monitoring and evaluation of savings; Maintenance and operation; Equipment supply; Provision of services (space heating/cooling, lighting, etc.); Fuel or electricity supply; Project financing etc. Consider using multiple agreements for the deployment of the SmartSPIN service if that simplifies the engagement with the client (such as a contract for installing smart metering, a contract for obtaining/monitoring energy consumption data etc).	The engagement with landlords and tenants will be simplified if they are introduced gradually to the SmartSPIN service.	Lack of trust in ESCO services. Conviction that ESCO services are complicated to understand, and their benefits unclear	Interview 9. The technical notes ( Técnico Lisboa, 2018).
18	Deliver the SmartSPIN service using a Performance Guarantee model	Several Landlords are not aware of the Performance Guarantee business model and are afraid of economic risks associated with renovation and installation of energy efficiency measures.	Lack of knowledge about ESCO business models.	Interview 8. SmartSPIN D2.2. ( Qin *et al*, 2017; Shang *et al*, 2017).
19	The SmartSPIN service must be based on a tripartite agreement between tenant, landlord and ESCO.	If the contractual agreement will be only established between landlord and ESCO then it is very likely that engagement of tenant will be modest.	Current contractual templates (e.g., EPC templates) enable to establish an agreement between ESCO and its client therefore are not suitable for the deployment of the SmartSPIN service.	Interview 8. Energy Performance Contracting model ( SEAI, 2022).
20	The SmartSPIN service should protect tenants from an increase of rent, which is not compensated by their higher energy efficiency. If a landlord wants to increase the rent due to additional benefits, the relevant documents and a calculation must be provided. Non-energy benefits should be delivered to tenants (such as improved comfort).	Tenants must be incentivized in agreeing to implement the SmartSPIN service. The service implementation cannot be fully successful if based only on a landlord’s decision.	The service is not attractive for tenants (and that will limit its uptake).	Interview 8. The article ( Zulliger, 2022).
21	Energy management systems of commercial buildings use weather forecasts (and others) to estimate energy savings. The SmartSPIN service should make use of forecasts of electricity market price and provide forecasts of energy savings and carbon emissions.	Currently many ESCOs are implementing software platforms including forecasting services. This is considered a necessary innovation to deliver a smart service and gain the trust of the clients.	Lack of accuracy in energy savings and carbon emissions predictions.	Interview 7. D2.2 and technical literature, e.g. ( Lazos *et al*., 2014)
22	The SmartSPIN service should be made compatible with innovative financing mechanisms including the schemes mentioned by the EU Directives (revolving funds, guarantees, and insurance schemes). Members of energy communities can use Crowdfunding to finance energy efficiency measures.	Lack of adequate financing mechanisms might discourage the acceptance of the SmartSPIN service.	Lack of adequate financing mechanisms. Inadequate policies. Lack of knowledge about bank's requirements for financing.	Interviews 7 and 10. Relevant H2020 projects in ( Zapfel, 2017). Financing mechanisms in ( Gallerand *et al*., 2022).
23	Multiple services may be provided from multiple types of community-level multi- energy flexibility. The most developed work on multi-service provision from demand-side resources focuses on electrical storage. In addition, similar work also exists for community- level multi-energy systems. The SmartSPIN service should include features related to demand-response, energy storage and energy flexibility to be competitive with new energy services that will conquer the market after 2024.	A forthcoming new version of the EU Energy Efficiency Directive will define the concept of expanded energy services and the offer of ESCOs will become aligned with that (2024 and beyond).	Lack of regulatory support for novel energy services based on demand response, energy storage and smart controls.	Interview 10. Scientific publications, e.g. ( Good & Mancarella, 2019).

## 5. Discussion

**Figure 9.**
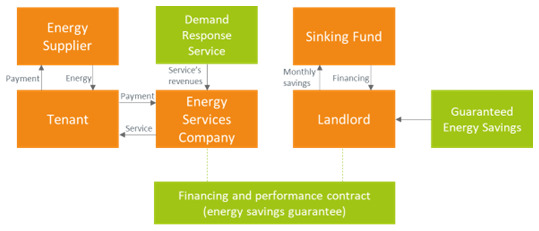
The SmartSPIN guaranteed savings business model.

The analysis of the business models provided in section 3, along with information gathered by means of the interviews of the stakeholders listed in section 2, has provided insights on the most suitable business model to demonstrate the alleviation of the split incentive issue in the commercial rented sector in Europe. It can be observed that in Ireland, Spain and Greece the ESCO markets are not mature yet. Table 4 shows the barriers to their development ( Bertoldi *et al*., 2019). Findings of ( Bertoldi *et al*., 2019) are also in agreement with our stakeholders’ analysis. Barriers to the ESCOs’ services obviously also hinder the adoption of the most advanced business models previously reviewed in the considered countries. It is unlikely that business models where an ESCO needs to liaise with other actors, such as another energy efficiency provider ( section 3.6, section 3.7, section 3.8) may be given full consideration by actors who lack experience and by potential clients who mistrust ESCOs. Therefore, it is unlikely that such business models will be successful in Ireland, Spain and Greece in the short term and will not be selected for SmartSPIN’s demonstration. With a maturation of the ESCO markets in Europe and in the considered countries, new market opportunities might arise when utility companies will co-create value connecting supply-side ESCOs with potential demand-side customers, keeping a customer-centric, service-dominant approach ( Badi, 2021). The comparative evaluation of the considered business models is presented in Table 5. The guaranteed savings model in section 3.3 seems the most suitable one to be adopted for the demonstration activities of the H2020 SmartSPIN project (with some changes). With such a model, the ESCO may concentrate on the achievement of energy savings and delivering them with performance guarantee. In relation to project financing, it was found that the pilot companies involved in the SmartSPIN project have established sinking funds by setting aside revenue over a suitable period of time to fund the future capital expense or repayment of a long-term debt for the implementation of energy efficiency measures. This facilitates the engagement and contracting with ESCOs that prefer to work with a guaranteed savings business model, leaving to their clients the responsibility of project funding. In fact, banks evaluate borrowers mainly on their creditworthiness, without being able to assess the actual capacity of an energy efficiency project to create energy savings and cash flows. The loan can be paid back by the cash flows generated by the project only in case of some large-scale projects ( Andaloro *et al*., 2022). In addition, a demand response service may be established and managed by the ESCO, which will contribute to add economic value to the energy savings achieved with the efficiency measures and in turn to a higher value shared with the clients ( Figure 9).

**Table 4.** ESCO market development in Ireland, Spain and Greece.

N.	Country	ESCO market development	Barriers
**1**	Ireland	Developing market: small or large size and/or growing	Lack of experience of actors; lack of appropriate forms of finance.
**2**	Spain		Small size of projects and high transaction costs; mistrust from the (potential) clients.
**3**	Greece	Embryonic market: small and/or non-growing	Lack of appropriate forms of finance; limited in-house technical expertise.

**Table 5.** Comparative evaluation of business models for the SmartSPIN energy service.

N.	Business model	Evaluation
**1**	Equipment lease	Too simple, does not allow to deliver a smart energy service based on multiple energy efficiency measures
**2**	Shared savings business model	Capital investment makes it more difficult for the ESCO to take on additional EPC projects
**3**	Guaranteed savings business model	A well-known and widely adopted model that can be extended to tackle the split incentive issue
**4**	Chaffee business model	It requires that the ESCO assume the risk of rising energy prices. Therefore, it can only be used by ESCOs with a strong technical capacity.
**5**	Finance lease business model	This model requires high-income return from the project's investment.
**6**	Energy efficiency as a service Business model	These business models are most likely unknown in the European ESCO markets. The stakeholders cannot see immediate benefits of these models based on their knowledge of the European markets. The barriers are lack of information, inexperience of actors, lack of facilitators, lack of a trusted method to monitor and verify energy savings, high transaction costs ( Bertoldi *et al*., 2019; Brown *et al*., 2022).
**7**	Managed energy services agreement model	
**8**	Metered energy efficiency transaction structure	

**Figure 10.**
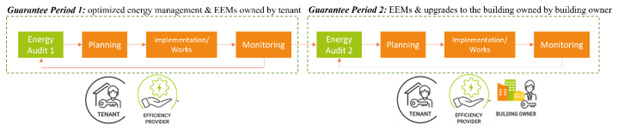
Proposal for a tripartite energy performance contract.

**Figure 11.**
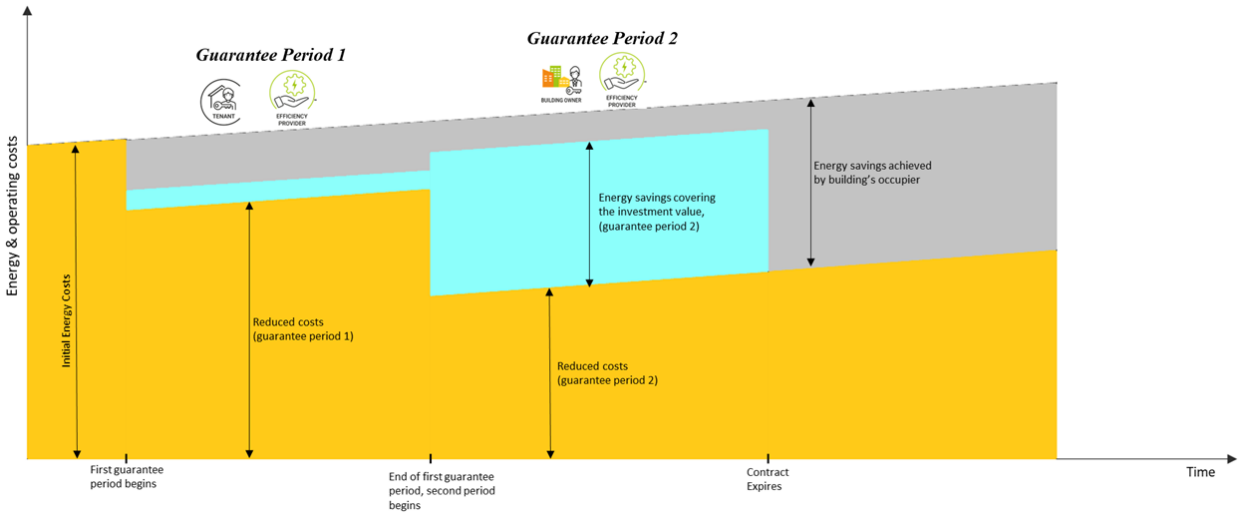
Energy savings model for a tripartite energy performance contract.

The smart energy service can be delivered in two steps. The first step requires the engagement of the tenant for the implementation of energy management measures and the installation of energy efficient equipment and energy efficiency measures (EEMs) owned by the tenant. The second step requires the engagement of the landlord and obtaining their consent for implementation of other EEMs and upgrades to the building which require substantial works. The service can be delivered using a tripartite EPC with two guarantee periods, if necessary ( Figure 10). The same steps characterize each period: an energy audit, the planning of EEMs, their implementation with upgrade works, the monitoring of performances and the possibility of repeating such steps if the desired performances are not achieved with the selected package of EEMs. It is foreseeable that the EEMs installed in the first step are simpler than those installed in the second step, therefore they result in a lower energy cost reduction and lower implementation costs ( Figure 11). The energy cost savings obtained with the first step (taken from the ESCO) can be partially used to finance the EEMs installed in the second step.

Finally, it is worth noting that the literature has shown that buildings’ occupants tend to use more energy than expected after retrofits, which also results in underestimated retrofitting costs. This is known as rebound effect. It was recommended in ( Lu *et al*., 2017) to reduce such a rebound effect by sharing part of the savings with the tenant. The paper presented a model to evaluate the Net-Present-Value of an energy efficiency project for the building owner at different contract durations and sharing percentages with tenant. However, the analysis presented in ( Lu *et al*., 2017) assumes that the rebound effect can be modelled by means of an exponential function called standard utility function. Standard utility function is described by three parameters, the risk tolerance (which determines the curvature of the function and can be used to differentiate risk attitudes of various renters) and two other constants defining the boundary conditions. The main limitation of the study is that the validation of the proposed model using data gathered from the field (including the correct estimation of its parameters) would require several campaigns to collect a large amount of data, which cannot be easily delivered by a single project. Moreover, tenants may have a low preference for green building features with respect to rental rate, convenience of tenant operations, safety and security the study ( Adnan *et al*., 2013). This indicates that sharing of savings with tenants is a necessary catalyst to stimulate their interest in green buildings and energy efficiency.

## 6. Conclusions

This paper has analyzed eight ESCO business models to deliver energy efficiency through smart energy services to the commercial rented sector and the driving factors, which may enable to overcome the split incentive issue and to enter in an effective tripartite EPC agreement involving building owner, tenant and ESCO. Furthermore, the features of a smart energy service suitable for the commercial sector have been investigated by means of interviews with key stakeholders and the literature. The proposed SmartSPIN service will comprise several features: energy management, change of the electricity supplier, equipment or renewable energy source installation ( *e.g*., solar PV installations) or replacement, installation of sensors, energy monitoring, operation and maintenance (O&M) services, providing advice on how to reduce energy consumption, etc. These features will be delivered ensuring benefits for all the involved parties. The SmartSPIN service will have to be delivered in a manner which will consider the existing relationship between landlord and tenant. If the landlord owns all the equipment installed in the rented unit, the ESCO could engage with landlord only. However, if tenants are allowed to install their own equipment and own some parts that can be replaced with more energy efficient counterparts, then the ESCO will engage with them as well. This is the most general case that must be addressed by the SmartSPIN service.

EaaS include five major benefits: energy management, maintenance, total guarantee of the equipment, improvement works, and improvement of energy efficiency. Exceptions may apply for specific cases and obviously, the service must be flexible to cover the cases where not all the mentioned benefits apply. A trusted and standardized Measurement and Verification (M&V) approach will be developed to determine the energy savings applicable to the various features of the SmartSPIN service. The M&V process must address all the concerns in relation to the level of uncertainty associated with the measurements. The lack of a trusted M&V process may become a barrier to the deployment of the energy efficiency service. The SmartSPIN service should include, where applicable, a dynamic tariff for the electricity, gas consumption and water used by the tenants, which is fair, easy to understand and independent of the season.

The SmartSPIN smart energy service will be delivered using a guaranteed savings business model, which has been adapted to the rented scenario to solve the split incentive issue. It is also proposed to adapt the standard EPC to the rented scenario for the commercial sector, establishing a tripartite agreement between landlord, tenant and ESCO which represents the legal/contractual framework required to deliver benefits deriving from energy efficiency to all the parties.

## Data Availability

No underlying data are associated with this article.
