# Supplementary material for: Analysis of business models for delivering energy efficiency through smart energy services to the European commercial rented sector
Source: Open Res Eur. 2024 Jan 15;2:131. Originally published 2022 Nov 28. [Version 2] doi: 10.12688/openreseurope.15240.2 (PMC10899787; doi:10.12688/openreseurope.15240.2)
Supplement: Peer review history

# Reviewer report: Reviewer response for version 2

**DOI:** 10.21956/openreseurope.18392.r37432

**Published:** 2024-02-27

**Authors:** Nichole Hanus

**Affiliations:** 1 Lawrence Berkeley National Laboratory, Berkeley, California, USA

Thank you for the considerable improvement in paper quality. The paper is far more readable and I appreciate the extension of the literature review, added details of the interviews, and explanation of the business model validation process.

Is the study design appropriate and does the work have academic merit?

Partly

Is the work clearly and accurately presented and does it cite the current literature?

Partly

If applicable, is the statistical analysis and its interpretation appropriate?

Not applicable

Are all the source data underlying the results available to ensure full reproducibility?

Partly

Are the conclusions drawn adequately supported by the results?

No

Are sufficient details of methods and analysis provided to allow replication by others?

No

Reviewer Expertise:

Energy efficiency investment decision-making

I confirm that I have read this submission and believe that I have an appropriate level of expertise to confirm that it is of an acceptable scientific standard.

# Reviewer report: Reviewer response for version 2

**DOI:** 10.21956/openreseurope.18392.r37431

**Published:** 2024-02-12

**Authors:** Mahyar Kamali Saraji

**Affiliations:** 1 Kaunas Faculty, Vilnius University, Kaunas, Lithuania

The authors have made substantial improvements to the article according to the comments. Therefore, the current form of the article is deemed suitable for indexing.

Is the study design appropriate and does the work have academic merit?

Partly

Is the work clearly and accurately presented and does it cite the current literature?

Partly

If applicable, is the statistical analysis and its interpretation appropriate?

Partly

Are all the source data underlying the results available to ensure full reproducibility?

Partly

Are the conclusions drawn adequately supported by the results?

Partly

Are sufficient details of methods and analysis provided to allow replication by others?

No

Reviewer Expertise:

Sustainability, Energy transition.

I confirm that I have read this submission and believe that I have an appropriate level of expertise to confirm that it is of an acceptable scientific standard.

# Reviewer report: Reviewer response for version 1

**DOI:** 10.21956/openreseurope.16482.r35409

**Published:** 2023-10-11

**Authors:** Mahyar Kamali Saraji

**Affiliations:** 1 Kaunas Faculty, Vilnius University, Kaunas, Lithuania

The present study conducted a comparative analysis of business models used by ESCOs, contributing to dealing with split-incentive issues to develop a new ESCO business model. The study also provided recommendations for enhanced energy service in the commercial sector, and the proposed model was implemented in Greece, Ireland, and Spain. The present study's main flaw is using the research method for comparative business models. The following comments would improve the acceptability of the present research.

**Abstract**

 Results should be presented briefly in the abstract.

**Introduction**

 Please dedicate a paragraph to contributions. The contributions of the study are not clear or hard to follow.

 The article's structure should be presented at the end of the introduction.

**Methods**

 Surprisingly, there is no clear information regarding the research steps. For instance, which features of business models were considered in comparative analysis? How did the authors interview the experts? How did the authors validate the collected data through interviews? Overall, the research method's steps should be explained clearly and followed quickly by readers. It is recommended to provide a flow chart illustrating the research steps. Also, the authors should explain why they implemented the proposed business model in Greece, Ireland, and Spain.

**Sections 3, 4, and 5**

 Well presented.

**Conclusions**

 Any limitations to the present study?

 Provide some accurate recommendations for future studies.

Is the study design appropriate and does the work have academic merit?

Partly

Is the work clearly and accurately presented and does it cite the current literature?

Partly

If applicable, is the statistical analysis and its interpretation appropriate?

Partly

Are all the source data underlying the results available to ensure full reproducibility?

Partly

Are the conclusions drawn adequately supported by the results?

Partly

Are sufficient details of methods and analysis provided to allow replication by others?

No

Reviewer Expertise:

Sustainability, Energy transition.

I confirm that I have read this submission and believe that I have an appropriate level of expertise to confirm that it is of an acceptable scientific standard, however I have significant reservations, as outlined above.

# Response

**Published:** 2023-12-17

**Authors:** Luciano De Tommasi

**Affiliations:** International Energy Research Center, Ireland

Dear reviewer, many thanks for your detailed and constructive comments. Please find below answers to each of them and text that has been added to the paper to address them. This can also be viewed in the file linked here.   

 The present study conducted a comparative analysis of business models used by ESCOs, contributing to dealing with split-incentive issues to develop a new ESCO business model. The study also provided recommendations for enhanced energy service in the commercial sector, and the proposed model was implemented in Greece, Ireland, and Spain. The present study's main flaw is using the research method for comparative business models. The following comments would improve the acceptability of the present research.   Thanks for your comment. We wish to clarify that the research method adopted for comparison of business models in our paper is participatory research. The following paragraph has been added to section 2 (methodology).   The research method used in this paper to compare business models for Energy Performance Contracting applicable to the European commercial rented sector is participatory research. Participatory research engages stakeholders to work with researchers through all stages of the research process, from developing the research questions to the final dissemination of results (Duea et al., 2022). Stakeholders previously engaged to provide feedback on project’s progress and outcomes were consulted to collect their feedback regarding the set of business models previously reviewed by the authors and their recommendation to implement the most appropriate one for the European commercial rented sector. The objective of participatory research was to explore the issue of the business model selection/identification from stakeholder perspective and to prioritize an action strategy for the energy efficiency service implementation.   The following reference has been added:   Duea, S. R., Zimmerman, E. B., Vaughn, L. M., Dias, S., & Harris, J. (2022). A Guide to Selecting Participatory Research Methods Based on Project and Partnership Goals. *Journal of Participatory Research Methods*, *3*(1). https://doi.org/10.35844/001c.32605

 Abstract

 Results should be presented briefly in the abstract.

   Thank you for your comment **.** The following sentence has been added to the abstract: Finally, it is argued that the classical shared savings and guaranteed savings ESCOs models may be adapted to the commercial rented sector and used at SmartSPIN demonstration sites in Spain, Greece and Ireland. The guaranteed savings model appears to be the most appropriate one to use when the building owner is funding the energy efficiency project using own funds or liaising directly with a bank or other finance provider.

 Introduction

 Please dedicate a paragraph to contributions. The contributions of the study are not clear or hard to follow.

 The article's structure should be presented at the end of the introduction.   Subsection 1.2 has been added to the introduction to describe the contributions and to present the article’s structure. 1.2 Contributions The contributions of this paper are two. First one is to discuss eight business models that can be used to deploy a smart energy service which can increase energy efficiency in commercial rented properties. Second one is to collect information from previously identified representative stakeholders that are used in the first instance to identify key recommendations to implement such smart energy service and in the second instance to determine the structure and characteristics of the business model most suitable for a demonstration in pilot sites located in Ireland, Spain and Greece. The structure of the article is as follows. After providing a comprehensive introduction to the subject matter in section 1, section 2 introduces the methodology used to perform the research described in the paper. Section 3 reviews eight business models relevant with the commercial rented sector. Section 4 discusses a list of recommendations identified by the research team considering the point of view of the stakeholders. Section 5 is a discussion that considers the business models of section 3 in the perspective of the stakeholders, identifying the most suitable business model to demonstrate the alleviation of the split incentive issue at the SmartSPIN project's pilot sites. Section 6 concludes the paper and summarises its findings.

 Methods

 Surprisingly, there is no clear information regarding the research steps. Thank you for your valuable comment. Section 2 “Methodology” has been updated including information regarding the research steps.   For instance, which features of business models were considered in comparative analysis?   Thank you for your comment. The following text has been added in section 2.1   In this paper, the business models have been compared considering their value proposition for the clients (i.e., considering what the client needs and/or fears) and how well they would fit within the environment (markets of smart energy services in Ireland, Spain and Greece) and stakeholders.   How did the authors interview the experts?   Thank you for your comment. The following text has been added in section 2.2:   The interview protocol consisted in an introduction to the project provided by the interviewer, followed by a semi-structured interview with some predetermined open questions that covered background information of the interviewee, best practices to implement energy efficiency projects, the challenges in the commercial private sector and the known limitations of current approaches. The goal of interviews was to determine a comprehensive set of recommendations that would provide a context to conduct further research in other tasks of the project on how to solve the split incentive issue. To facilitate the participation of stakeholders, the interviews were conducted orally and it was not required that stakeholders would prepare any answers to interview questions in advance of the interview. The interviews were audio-recorded with the consent of the interviewees and the transcriptions (obtained via software) were thoroughly analysed to determine the recommendations for an enhanced energy service in the commercial sector included in this paper.   How did the authors validate the collected data through interviews?   Thank you for your comment. The following text has been added to section 2.2: Data collected through interviews have been validated against the published literature (Table 3).   Overall, the research method's steps should be explained clearly and followed quickly by readers. It is recommended to provide a flow chart illustrating the research steps. Thank you for your comment. The research method’s steps have been explained in section 2.1 and a flow chart illustrating the research steps is provided in Figure 1.   Also, the authors should explain why they implemented the proposed business model in Greece, Ireland, and Spain. Thank you for your comment. The following text has been added in section 5 (Discussion) to clarify the point raised by the reviewer. It can be observed that in Ireland, Spain and Greece the ESCO markets are not mature yet. For this reason, these countries show a large potential to uptake novel smart energy services that leverage the business model identified in this study. Sections 3, 4, and 5

 Well presented. Thank you for your positive comment.

 Conclusions

 Any limitations to the present study?   Thank you for your comment. Limitations have been added to the conclusions. The following text has been added to section 6:

   The limitation of the proposed study is mainly related to the number of representative stakeholders that were enrolled in the research (sample size) and the protocol used for the interviews that aimed to facilitate the enrollment of stakeholders in the study (oral interviews where the interviewees did not receive the questions before the interview and therefore were not asked to prepare their answers in advance). Moreover, the study covered only Ireland, Spain and Greece.   Provide some accurate recommendations for future studies.   Thank you for your comment. The following text has been added to section 6:

   As for future recommendations, it is observed that the presented study could be replicated in other European countries following steps similar to those illustrated in Figure 1. Moreover, once that the business model/smart energy service is implemented at selected pilot sites, additional information can be collected from the stakeholders to highlight practical implementation issues and possible workarounds.

# Reviewer report: Reviewer response for version 1

**DOI:** 10.21956/openreseurope.16482.r34396

**Published:** 2023-09-06

**Authors:** Nichole Hanus

**Affiliations:** 1 Lawrence Berkeley National Laboratory, Berkeley, California, USA

**General Comments:**

 This is an interview study comparing and contrasting eight ESCO business models, aimed at the goal of overcoming split incentive issues in the commercial rented sector across Europe. After qualitative discussion of the benefits and barriers to each ESCO business model, the authors validated the models in three demonstration sites: Spain, Greece, and Ireland. Overall, this paper could be improved by a more rigorous assessment of the interview data. Furthermore, the “validation” method for the three demonstration sites also lacks empirical grounding and the resulting recommendations for an enhanced energy service in the commercial sector are tenuous. I believe this paper needs substantial improvements before publication.

 My general comments follow, preceding my section-by-section comments.

  

- There are some inconsistencies in tense use (e.g., switching from present to past tense in a paragraph).
- The paper could use a final check for grammar and spelling (e.g., “They have been obtained from a detailed analysis of ten interviews of key stakeholders of the energy efficiency section and of the commercial rented sector…” It should read “energy efficiency sector”).

 

- **Abstract**
- I would explain the validation method in the abstract. It currently reads as though the paper is referencing existing projects in Spain, Greece, and Ireland – rather than hypotheticals.

 

- **Introduction:**
- The authors might consider providing a bit more context about how energy investments are often studied at the individual- or organization-level. These two contexts aren’t exactly described in this literature review. Furthermore, the split-incentive issue sometimes arises in the middle of the Venn-Diagram (e.g., individual renters butting up against building managers/owners).
- Here are some references I might consider in the introduction:
  - Individual EE decision-making:
    - Theory of Planned Behavior:
      - I. Ajzen (1991). The theory of planned behavior ^1^.
      - P. C. Stern (2000). Toward a Coherent Theory of Environmentally Significant Behavior ^2^.
    - Information Deficit Model
      - P. Sturgis and N. Allum (2004). Science in Society: Re-Evaluating the Deficit Model of Public Attitudes ^3^.
      - P. S. Hart and E. C. Nisbet (2012). Boomerang Effects in Science Communication ^4^.
    - Theory of Motivated Reasoning
      - Z. Kunda (1990). The case for motivated reasoning ^5^.
      - C. S. Taber and M. Lodge (2006). Motivated Skepticism in the Evaluation of Political Beliefs ^6^.
  - Organizational EE decision-making:
    - Capital Investment Theory
      - S. T. Anderson and R. G. Newell (2004). Information programs for technology adoption ^7^.
      - A. B. Jaffe and R. N. Stavins (1994). The energy-efficiency gap – What does it mean? ^8^
    - Organizational Behavior Theory
      - S. Sorrell et al. (2000). Reducing barriers to energy efficiency in public and private organisations ^9^.
      - C. Cooremans (2011). Make it strategic! Financial investment logic is not enough ^10^.

 

- **Methods:**
- The methods section could be broken into two sub-sections: (1) describing the interview sample, interview protocol, and data analysis methods; and (2) the framework for analyzing the ESCO business models.
- The current methods section is too short and doesn’t provide enough empirical grounding for the rest of the paper.

 

- **Business models for the commercial rented sector addressing the split-incentive issue:**
- The individual flow-charts are very helpful.

 

- **Recommendations for an enhanced energy service in the commercial sector:**
- Although the list of recommendations is comprehensive, the lack of description for the interview protocol or corresponding data coding leads to these recommendations feeling very high-level and not grounded in systematic findings from the interviews.

 

- **Discussion:**
- The discussion begins with the assumption that the reader understands how the authors did the, “analysis of the business models provided in section 3...” This is not true. Rather, the reader has no conceptual understanding of how the authors did this down-selection or mapping.

 

- **Conclusions:**
- Once the issues above are addressed, I believe the Conclusion will need to be re-written.

Is the study design appropriate and does the work have academic merit?

Partly

Is the work clearly and accurately presented and does it cite the current literature?

Partly

If applicable, is the statistical analysis and its interpretation appropriate?

Not applicable

Are all the source data underlying the results available to ensure full reproducibility?

Partly

Are the conclusions drawn adequately supported by the results?

No

Are sufficient details of methods and analysis provided to allow replication by others?

No

Reviewer Expertise:

Energy efficiency investment decision-making

I confirm that I have read this submission and believe that I have an appropriate level of expertise to confirm that it is of an acceptable scientific standard, however I have significant reservations, as outlined above.

## References

[1] : The theory of planned behavior. *Organizational Behavior and Human Decision Processes* .1991;50(2) : 10.1016/0749-5978(91)90020-T 179-211 10.1016/0749-5978(91)90020-T

[2] : New Environmental Theories: Toward a Coherent Theory of Environmentally Significant Behavior. *Journal of Social Issues* .2000;56(3) : 10.1111/0022-4537.00175 407-424 10.1111/0022-4537.00175

[3] : Science in Society: Re-Evaluating the Deficit Model of Public Attitudes. *Public Understanding of Science* .2004;13(1) : 10.1177/0963662504042690 55-74 10.1177/0963662504042690

[4] : Boomerang Effects in Science Communication. *Communication Research* .2012;39(6) : 10.1177/0093650211416646 701-723 10.1177/0093650211416646

[5] : The case for motivated reasoning. *Psychol Bull* .1990;108(3) : 10.1037/0033-2909.108.3.480 480-98 10.1037/0033-2909.108.3.480 2270237

[6] : Motivated Skepticism in the Evaluation of Political Beliefs. *American Journal of Political Science* .2006;50(3) : 10.1111/j.1540-5907.2006.00214.x 755-769 10.1111/j.1540-5907.2006.00214.x

[7] : Information programs for technology adoption: the case of energy-efficiency audits. *Resource and Energy Economics* .2004;26(1) : 10.1016/j.reseneeco.2003.07.001 27-50 10.1016/j.reseneeco.2003.07.001

[8] : The energy-efficiency gap What does it mean?. *Energy Policy* .1994;22(10) : 10.1016/0301-4215(94)90138-4 804-810 10.1016/0301-4215(94)90138-4

[9] : Reducing barriers to energy efficiency in private and public organisations. Final Report. *Fraunhofer-Publica* .2000; Reference source

[10] : Make it strategic! Financial investment logic is not enough. *Energy Efficiency* .2011;4(4) : 10.1007/s12053-011-9125-7 473-492 10.1007/s12053-011-9125-7

# Response

**Published:** 2023-12-17

**Authors:** Luciano De Tommasi

**Affiliations:** International Energy Research Center, Ireland

Dear Reviewer, many thanks for your detailed and constructive comments to our paper. Your remarks are answered one by one below and the corresponding changes applied to the manuscript are highlighted in yellow - this can be viewed in the file linked here.

 General Comments:

 This is an interview study comparing and contrasting eight ESCO business models, aimed at the goal of overcoming split incentive issues in the commercial rented sector across Europe. After qualitative discussion of the benefits and barriers to each ESCO business model, the authors validated the models in three demonstration sites: Spain, Greece, and Ireland. Overall, this paper could be improved by a more rigorous assessment of the interview data. Furthermore, the “validation” method for the three demonstration sites also lacks empirical grounding and the resulting recommendations for an enhanced energy service in the commercial sector are tenuous. I believe this paper needs substantial improvements before publication. Thank you for your constructive comments. The paper has been thoroughly revised carefully addressing all your comments. It is clarified that interview data were thoroughly assessed by analyzing transcriptions of interviews; additional information included in the paper that clarify the interview sample and the protocol highlight the procedure followed to obtain the recommendations included in section 4. List of recommendations has been revised in version 2 of the paper. It is remarked that this paper focuses on the analysis of business models and down-selection of the model that best applies to Ireland, Spain and Greece in view of its final validation at the three project’s demonstration sites. Final validation of the smart energy service at the sites is currently in progress and is out of the scope of this paper though. The methodology section has been expanded to provide a better empirical grounding for the paper. My general comments follow, preceding my section-by-section comments. There are some inconsistencies in tense use (e.g., switching from present to past tense in a paragraph). Thanks for your comment; the paper has been revised to remove such inconsistencies.   The paper could use a final check for grammar and spelling (e.g., “They have been obtained from a detailed analysis of ten interviews of key stakeholders of the energy efficiency section and of the commercial rented sector…” It should read “energy efficiency sector”). Thank you for your comment; the final check has been done and the typo that you indicated has been corrected. **Abstract** I would explain the validation method in the abstract. It currently reads as though the paper is referencing existing projects in Spain, Greece, and Ireland – rather than hypotheticals. Thanks for your comment. The following sentence is added to the abstract to explain the validation method: The validation method for the comparative analysis of business models and selection of the most appropriate one is based on both literature review and consultation of selected stakeholders’ (stakeholder value creation framework). The research steps followed in this study are illustrated in Figure 1. The sentence that references the projects has been modified to clarify that they are not hypothetical but on going at the time of writing. However, detailed description of the concrete projects is out of the scope for this paper, which is a review of business models and recommendations supported by stakeholders’ interviews. The sentence is now modified as follows: The pilot implementation of project is in progress in a business park in Greece, in an office building in Ireland and in two shopping centers in Spain. **Introduction:** The authors might consider providing a bit more context about how energy investments are often studied at the individual- or organization-level. These two contexts aren’t exactly described in this literature review. Furthermore, the split-incentive issue sometimes arises in the middle of the Venn-Diagram (e.g., individual renters butting up against building managers/owners). Thank you for your comments. The following text has been added to the introduction: Energy investments are often studied considering how the related decision-making occurs at a variety of levels in organisations. Most of the organisations follow a bottom-up  procedure, that requires final approval from an individual who has financial authority, such as the building owner, the company's CEO or CFO. The decisions regarding equipment selection are often made by contractors rather than by managers, especially in small-sized organisations. Equipment utilization decisions are usually made by individuals that are part of the organisation that implements energy efficiency measures in its premises. In rented properties, building owners consider tenant retention and attraction (which would be determined by a reduction in their operational energy costs) important aspects when making investment decisions in energy efficiency measures. Moreover, decision makers tend to avoid energy-efficiency investments that could compromise health and comfort of tenants (Parker et al., 2000). The risk of individual renters butting up against building managers/owners could be reduced in the commercial sector by setting up common goals for energy efficiency and sustainability, which can be agreed as part of a green lease. Green leases are an appropriate tool to establish win-win relationships between renters and building owners. In fact, higher energy efficiency of a property increases its market value and demand by most of tenants (benefit for building owner), whereas it reduces the operational energy costs for renters and contribute toward achieving their own sustainability goals (benefits for renters). The following reference has been added: Parker, G., Chao, M., & Gillespie, K. (2000). Energy-related practices and investment criteria of corporate decision makers. In Proceedings of the 2000 summer study on energy efficiency in buildings. Washington, DC: American Council for an Energy Efficient Economy. Here are some references I might consider in the introduction: Thanks for suggesting references. All the references suggested by the reviewer have been added. The sentences to introduce them are provided below highlighted in yellow. Individual EE decision-making: Theory of Planned Behavior: Ajzen (1991). The theory of planned behavior. According to the theory of planned behavior (Ajzen, 1991) behavioral achievement depends jointly on motivation (intention to perform a given behavior, e.g., investing in energy efficiency) and ability (behavioral control). P. C. Stern (2000). Toward a Coherent Theory of Environmentally Significant Behavior. When it comes to undertaking environmental significant behaviors (such as engaging in the decarbonization of the commercial rented sector), several causal variables, such as attitudinal, personal capabilities, contextual factors, habit and routine, may influence these behaviors (Stern, 2000). Information Deficit Model   P. Sturgis and N. Allum (2004). Science in Society: Re-Evaluating the Deficit Model of Public Attitudes. Like in other fields of science, a complex range of circumstances surrounding scientific and technological development within a wider political framework determine the development of effective solutions to improve energy efficiency in the rented sectors (Sturgis and Allum, 2004). P. S. Hart and E. C. Nisbet (2012). Boomerang Effects in Science Communication. Finally, the importance of communicating how energy efficiency and decarbonisation of the built environment can combat climate change must be recognised. Indeed, if the general public had more information on climate change, they would be encouraged to adopt opinions consistent with those of experts (Hart and Nisbet, 2012).   Theory of Motivated Reasoning   Z. Kunda (1990). The case for motivated reasoning. On the other hand, if decision-makers cannot eventually act according to their beliefs (e.g., because of existing barriers) they will likely change them to reduce the dissonance between actions and beliefs (Kunda, 1990). C. S. Taber and M. Lodge (2006). Motivated Skepticism in the Evaluation of Political Beliefs.

 On the other hand, people may also have difficulties evaluating evidence because they are usually influenced by their prior feelings and biases. Such “prior attitude effect” should be carefully considered during service validation, when relevant people are asked to provide feedback on the service (e.g., by means of surveys or interviews). They should be instructed as much as possible to "put their feelings aside", "evaluate arguments fairly" and "be as objective as possible" (Taber and Lodge, 2006). Organizational EE decision-making:   Capital Investment Theory   S. T. Anderson and R. G. Newell (2004). Information programs for technology adoption. The role of service provider (such as an ESCO) includes the provision of consultancy and guidance to their clients regarding the technologies available on the market. In fact, payback, cost, savings, energy prices, and energy conservation quantities, do not fully explain the client’s technology adoption decision. If such variables are held constant, it has been observed that certain types of technologies and projects are more likely to be adopted than others (Anderson and Newell, 2004).

 A. B. Jaffe and R. N. Stavins (1994). The energy-efficiency gap – What does it mean? Furthermore, service providers may effectively contribute to eliminating market failures of energy technologies and reaching their full economic potential by providing relevant information to those subjects who may be interested in adopting them (Jaffe and Stavins, 1994).   Organizational Behavior Theory S. Sorrell et al. (2000). Reducing barriers to energy efficiency in public and private organisations. Moreover, the energy efficiency service eliminates the barrier of hidden costs (in addition to the split incentives), such as overhead costs for management, disruption, inconvenient, staff training, as well as other costs related to gathering and analysis of information (Sorrell et al., 2000), because the relevant management, maintenance and training tasks are performed by the service provider.  C. Cooremans (2011). Make it strategic! Financial investment logic is not enough. However, financial factors only partially determine decisions in investing in energy efficiency. In fact, organizational factors, such as organizational energy culture, power relationships, managers’ interests and mindsets, may determine firms’ behaviors that deviate from the rational behavior of economic actors aiming to profit maximization. A smart energy service may create strategic resources such as a comfortable indoor environment, where heating, cooling and ventilation systems are efficiently operated, as well as a positive image and reputation of the company (Cooremans, 2011). Risk reduction provided by an EPC (such as credit risk and performance risk) may tangibly reduce the risk barrier (Sorrell et al., 2000), triggering investment decisions, and generate a competitive advantage for the firms (Cooremans, 2011). **Methods:** The methods section could be broken into two sub-sections: (1) describing the interview sample, interview protocol, and data analysis methods; and (2) the framework for analyzing the ESCO business models. Thank you for your valuable comment. The suggestion has been implemented in the revised version of the paper. The interview sample consisted in ten stakeholders whose profile is listed in section 2. The following text has been added to clarify what the sample is: The profiles of the stakeholders selected for an interview (interview sample) are: The following text has been added to clarify the interview protocol that led to the recommendations included in the paper. The interview protocol consisted in an introduction to the project provided by the interviewer, followed by a semi-structured interview with some predetermined open questions that covered background information of the interviewee, best practices to implement energy efficiency projects, the challenges in the commercial private sector and the known limitations of current approaches. The goal of interviews was to determine a comprehensive set of recommendations that would provide a context to conduct further research in other tasks of the project on how to solve the split incentive issue. The following text has been added to clarify the data analysis method. To facilitate the participation of stakeholders, the interviews were conducted orally and it was not required that stakeholders would prepare any answers to interview questions in advance of the interview. The interviews were audio-recorded with the consent of the interviewees and the transcriptions (obtained via software) were carefully analysed by the authors taking notes that have been used to write the recommendations for an enhanced energy service in the commercial sector included in this paper. The following text has been added to clarify the framework for analyzing the ESCO business models: The framework used to analyse the ESCO business models is the stakeholder value creation framework (Freudenreich et al., 2020). This framework aims to create value with and for stakeholders when determining a business model. The authors held a consultation with the stakeholders to present the business models reviewed in section 3, and obtain their feedback. A group of representative stakeholders attended a first online meeting, then were consulted for individual interviews (section 4) then a final consultation was held via an online meeting to determine the characteristics of the desired business model. The following reference has been added: Freudenreich, B., Lüdeke-Freund, F., & Schaltegger, S. (2020). A stakeholder theory perspective on business models: Value creation for sustainability. Journal of Business Ethics, 166, 3-18. The current methods section is too short and doesn’t provide enough empirical grounding for the rest of the paper. Thanks for your constructive comment; the section has been expanded to address your previous comments and provide a better empirical grounding for the rest of the paper. **Business models for the commercial rented sector addressing the split-incentive issue:**

 The individual flow-charts are very helpful. Thanks for your positive comment. **Recommendations for an enhanced energy service in the commercial sector:**

   Although the list of recommendations is comprehensive, the lack of description for the interview protocol or corresponding data coding leads to these recommendations feeling very high-level and not grounded in systematic findings from the interviews. The following text has been added to clarify the interview protocol that led to the recommendations included in the paper. The interview protocol consisted in an introduction to the project provided by the interviewer, followed by a semi-structured interview with some predetermined open questions that covered background information of the interviewee, best practices to implement energy efficiency projects, the challenges in the commercial private sector and the known limitations of current approaches. The goal of interviews was to determine a comprehensive set of recommendations that would provide a context to conduct further research in other tasks of the project on how to solve the split incentive issue. To facilitate the participation of stakeholders, the interviews were conducted orally and it was not required that stakeholders would prepare any answers to interview questions in advance of the interview. The interviews were audio-recorded with the consent of the interviewees and the transcriptions (obtained via software) were carefully analysed by the authors taking notes that have been used to write the recommendations for an enhanced energy service in the commercial sector included in this paper. It is remarked that the purpose of the interviews was to collect the views of the stakeholders in relation to an enhanced energy efficiency service for the commercial rented sector, which contribute to the main objective of the paper, which is to review the business models in section 3 and to present them to the stakeholders to obtain the recommendations on the business model to be selected (section 5). Data coding occurred by analyzing the transcriptions of the interviews (obtained automatically by means of using a suitable software) and taking notes of the aspect that would be used to formulate a recommendation. **Discussion:**

 The discussion begins with the assumption that the reader understands how the authors did the, “analysis of the business models provided in section 3...” This is not true. Rather, the reader has no conceptual understanding of how the authors did this down-selection or mapping.   Thank you for your valuable comment. In the revised version of the paper (version 2) the steps followed to do the analysis of the business models provided in section 3 have been illustrated in Figure 1 included in section 2.1. The analysis was performed using the framework for analyzing the ESCO business models previously discussed in section 2. The following text has been added in section 2. The framework used to analyse the ESCO business models is the stakeholder value creation framework (Freudenreich et al., 2020). This framework aims to create value with and for stakeholders when determining a business model. The authors held a consultation with the stakeholders to present the business models reviewed in section 3, and obtain their feedback. Barriers, drivers and opportunities were discussed with stakeholders considering current market development conditions in Spain, Ireland and Greece. A group of representative stakeholders attended a first online meeting, then they were consulted for individual interviews (section 4) then a final consultation was held via an online meeting to determine the characteristics of the desired business model. The following reference has been added: Freudenreich, B., Lüdeke-Freund, F., & Schaltegger, S. (2020). A stakeholder theory perspective on business models: Value creation for sustainability. Journal of Business Ethics, 166, 3-18. In addition, the following text has been added in section 5 The analysis of the business models provided in section 3. has been conducted using the stakeholder value creation framework as described in section 2., involving representative stakeholders. **Conclusions:**

   Once the issues above are addressed, I believe the Conclusion will need to be re-written. The conclusions have been thoroughly rewritten considering the changes that have been applied to the paper.
